# Goat *SNX29*: mRNA expression, InDel and CNV detection, and their associations with litter size

**DOI:** 10.3389/fvets.2022.981315

**Published:** 2022-08-10

**Authors:** Qian Wang, Yi Bi, Zhiying Wang, Haijing Zhu, Mei Liu, Xianfeng Wu, Chuanying Pan

**Affiliations:** ^1^Key Laboratory of Animal Genetics, Breeding and Reproduction of Shaanxi Province, College of Animal Science and Technology, Northwest A&F University, Shaanxi, China; ^2^Shaanxi Provincial Engineering and Technology Research Center of Cashmere Goats, Yulin University, Yulin, China; ^3^Life Science Research Center, Yulin University, Yulin, China; ^4^College of Animal Science and Technology, Hunan Agricultural University, Changsha, China; ^5^Institute of Animal Husbandry and Veterinary, Fujian Academy of Agricultural Sciences, Fuzhou, China

**Keywords:** sorting nexin 29 (SNX29), mRNA expression, insertion/deletion (INDEL), copy number variation (CNV), goats, litter size

## Abstract

The sorting nexin 29 (*SNX29*) gene, a member of the SNX family, is associated with material transport and lipid metabolism. Previous studies have shown that lipid metabolism affects reproductive function in animals. Thus, we hypothesized there is a correlation between the *SNX29* gene and reproductive trait. To date, studies on the relationship between the *SNX29* gene and reproductive traits are limited. Therefore, the purpose of this study was to examine the polymorphism in the *SNX29* gene and its correlation with litter size. Herein, the mRNA expression levels of *SNX29* were assayed in various goat tissue. Surprisingly, we found that *SNX29* was highly expressed in the corpus luteum, large and small follicles. This result led us to suggest that the *SNX29* gene has a critical role in reproduction. We further detected potential polymorphisms in Shaanbei white cashmere (SBWC) goats, including insertion/deletion (InDel, *n* = 2,057) and copy number variation (CNV, *n* = 1,402), which were related to fertility. The 17 bp deletion (n = 1004) and the 20 bp deletion (*n* = 1,053) within the *SNX29* gene were discovered to be significantly associated with litter size (*P* < 0.05), and individuals the ID genotype of P1-Del-17 bp and the DD genotype of P2-Del-20bp had larger litter size. Additionally, the four CNV loci had significant correlations with litter size (*P* < 0.01) in our detected population. In CNV5, individuals with the median genotype were superior compared to those with loss or gain genotype in term of litter size, and in other three CNVs showed better reproductive trait in the gain genotype. Briefly, these findings suggest that *SNX29* could be used as a candidate gene for litter size in goat breeding through marker-assisted selection (MAS).

## Introduction

Litter size, one of the most important economic traits, is affected by multiple factors, among which genetic aspects are the most essential factors. However, so far there are few studies that identified the key genes affecting litter size (1). This requires us to develop modern molecular techniques, in order to find candidate genes that affect female fecundity. As a method of modern molecular breeding, marker-assisted selection (MAS) has plenty of advantages, such as efficiency, stability and reliability of outcomes, and good repeatability (2). Meanwhile, as a typical type of molecular marker, insertion/deletions (InDels) and copy number variations (CNVs) have been used in animal genetics and breeding (3), gene localization and gene cloning (4). InDel has been widely used in MAS, whereas CNV has been rarely utilized in breeding and has received attention in recent years.

The sorting nexin (SNX) family has more than 30 members, which are located in the cytoplasm and have membrane binding potential either binding to the PX domain through their lipids or through interactions between proteins and membrane-associated protein complexes (5, 6). This family is characterized by the involvement of the PX domain in cell-related functions (7), such as cell signaling and vesicle trafficking (8). Studies have shown that *SNX24* has a certain regulatory effect on estrogen (9). The sorting nexin 29 (*SNX29*) gene is one of the members of the SNX family and can bind to phosphatidylinositol. It has a similar protein domain and function as the *SNX24* gene (10). Previous studies have demonstrated that lipid metabolism affects reproductive function in animals (11), DNA methylation statuses of the *SNX29* gene were under the testosterone control (12). Thus, we speculated that the *SNX29* gene is an important candidate gene for reproductive trait.

Moreover, the relevant literatures describe that the *SNX29* gene has been proved to influence the formation of autism (13) and the regulation of nervous system development (14). Earlier researches have also reported that the *SNX29* gene is one of the translocation partners involved in the major histocompatibility complex class II transactivator *CIITA* (*MHC2TA*) in human KH-H2 cells (15) and is strongly associated with schizophrenia (16), intelligence (17), bipolar disorder (BPD) as well as major depressive disorder (MDD) (18). In addition, the *SNX29* gene has been identified as a growth-related gene that was correlated with body size and body weight (19), and it plays a vital role in York pigs (20) and other species. Growth and reproduction are inextricably linked. Most studies have revealed that genes can simultaneously affect growth traits and reproductive traits in livestock, such as the *PLAG1* gene (21–23). However, the effect of *SNX29* on reproductive traits of goats has not been reported at present, so we assumed that the *SNX29* gene is involved in reproduction.

Although *SNX29* has been widely studied with respect to neurodevelopment and growth traits, relevant studies on the relationship between the *SNX29* gene polymorphisms and reproductive traits are limited. Therefore, this study aimed to assay the mRNA expression level of *SNX29*, explore the genetic variations of the *SNX29* gene in Shaanbei white cashmere (SBWC) goats by polymerase chain reaction (PCR) and quantitative PCR (qPCR) detecting system, and verify its effect on reproductive traits. These results will help us to further study the relationship in livestock reproductive traits, and provide a new method to advance animal genetic reproduction and breeding, in order to improve the DNA molecular breeding technology in SBWC goats.

## Materials and methods

### Animal welfare explanation

All the samples in this experiment were in accordance with the use and management of experimental animals at Northwest A&F University (NWAFU-314020038).

### Sample collection

#### Total RNA extraction and examination

SBWC goats were randomly selected from the Yulin goat yard in Shaanxi province and were raised under the same feeding, water supply, and management conditions. Additionally, tissue samples including heart, liver, skeletal muscle, small follicles, large follicles, spleen, kidney, epididymis, testis, brain, fat, skin, ovary, and corpus luteum of adult SBWC goats were collected (*n* = 3) (24). The total RNA was extracted using Trizol reagent (Takara, Dalian, China) (25). The OD_260/280_ ratio was measured by a NanoDrop™1000 spectrophotometer (Thermo Scientific, Waltham, MA, USA) and a ratio between 1.8 and 2.0 meant qualified (26). Then, cDNA was synthesized using the PrimeScript RT Reagent Kit (Takara, Dalian, China) and temporarily stored at −20°C.

#### Genomic DNA isolation and DNA pool construction

Under the same feeding conditions, a total of 2,057 healthy SBWC goats were selected from Yulin goat farm in Shaanxi province and their ear tissues were collected. Using the phenol-chloroform method (27), genomic DNA was extracted from the goat ear tissues preserved in 70% alcohol at −80°C (28). OD values and OD_260/280_ ratios of DNA samples were measured by a NanoDrop™1000 Spectrophotometer (Thermo Scientific, Waltham, MA, USA). After quality detection, the DNA was diluted to 20 ng/μl and stored at −40°C for long-term preservation. After that, 30 samples were randomly selected to construct a pool of genomic DNA mixture to determine the presence of InDel sites occurred in the tested population.

### Primer design

We searched the Ensembl (https://asia.ensembl.org/) and Animal Omics database (http://animal.nwsuaf.edu.cn/), and found 11 InDel and five CNV loci of the *SNX29* gene in goats. Then, 16 primer pairs for amplification were designed using the Primer-BLAST tool in the NCBI database (https://www.ncbi.nlm.nih.gov/tools/primer-blast/) and the primer premier 5.0 software (Thermo Scientific, Waltham, MA, USA) (Table 1). Besides, four pairs of primers including P1-Del-17bp (Rs659002477, TAAAGGAAAGCAATGTA/-), P2-Del-20bp (Rs654310334, AGCTTCCGGTGAGCCTGTCG/-) (30), MC1R, and GAPDH (30, 31) were referred to our previous studies. *MC1R* and *GAPDH* were used as reference genes.

**Table 1:** The primer information of InDel, CNV and mRNA expression.

**Primer names**	**Primer pairs (5^′^-3^′^)**	**Tm (°C)**	**Length (bp)**	**Note**
P1-Del-17bp	F: TGGGCACATACATTTCAGGAC	58.28	144/127	Cited from:
	R: AAGCTCCCAAAGTCAGGAACC	60.20		26
P2-Del-20bp	F: GAGGAGATAAAGGGAGGGAGTC	58.00	199/179	Cited from:
	R: AGAAAGGCAGGCAGGTAAGG	58.80		26
P3-Del-20bp	F: CCTCGAAACCTTGGTCCATTC	58.92	299/279	This study
	R: ACACTCATCTTGCTCCTTTCCT	59.36		
P4-Del-18bp	F: GCTTCCTGGCTTGTTCTC	52.40	198/180	This study
	R: CTTGTTTCCGAACGATGA	51.70		
P5-Del-15bp	F: AGACCAAACAAACAGACAAACACA	59.72	147/132	This study
	R: TTTCAGAGCCTGGATACGCC	59.82		
P6-Del-20bp	F: TGGAGATTAAAGAGCGTGAGC	58.11	139/119	This study
	R: GGCCTCAAAGTTCCTTCACG	59.12		
P7-Del-18bp	F: AGATGGCAAATGAAAGTGCAGG	59.77	145/127	This study
	R: CCAGGCTTCTCTGTCTGTGGA	61.44		
P8-In-16bp	F: CTTCCATCAGCTCTGGTCACT	59.45	132/148	This study
	R: TGAAAGGATGGAGGCTAGGA	57.43		
P9-Del-15bp	F: TGCACAGTAGAGCTGGCAGT	61.48	109/94	This study
	R: CAAGAACCACAGACCCATCCA	59.93		
P10-Del-18bp	F: ACTGAATGTAGTTCCCTGTGC	57.94	189/171	This study
	R: ATGTGATGTGTGGCAGAACC	58.46		
P11-In-8bp	F: GTAACAATGCCACCCCCGA	60.00	109/117	This study
	R: GCCATCTTTCACCTGATGGGT	60.34		
CNV1	F1: TGTGGTGTGCAGGCTTCTTG	56.70	137	This study
	R1: CACGGGTTTGATCTCCAGTCC	56.60		
	F2: GAGCACGCTGCCTCATACATTG	58.40	101	This study
	R2: GGCGAGGGTGGGATGATTTGA	58.30		
CNV2	F: GGTCTGTGCTTAGTTGCTGTCC	57.60	138	This study
	R: TGGGTCGGGAAGATCCTTTGG	57.60		
CNV3	F: CGACTGAGCGACTTTCCCTTTC	57.70	104	This study
	R: AACCGAGACGACGCTGAGAC	57.90		
CNV4	F: GCCGCTGCTTTCTCTAACACAT	57.70	151	This study
	R: GCACCGCAACTTCTTCCTACTC	57.70		
CNV5	F: GAGGTGACATCGGTGCTCAGT	58.00	109	This study
	R: TGCCTGCTCCTGGAAGAGAATG	58.40		
MC1R	F: GGCCTGAGAGGGGAATCACA	61.27	126	Cited from:
	R: AGTGGGTCTCTGGATGGAGG	60.33		Bi et al. (29)
SNX29-mRNA	F: TCCTCTGAGCAGCCTGTTACCT	62.83	123	This study
	R: AGCGACCTGTTCTCTTCCTCCA	63.07		
GAPDH	F: AAAGTGGACATCGTTGCCAT	60.04	116	Cited from:
	R: CCGTTCTCTGCCTTGACTGT	59.97		Bi et al. (29)

*No specificity was detected in CNV1-F2 and CNV1-R2*.

### The mRNA expression levels of the *SNX29* gene

The relative mRNA expression levels of the *SNX29* gene was assayed by performing qPCR. The reaction system contained 5 μl of 2 × SYBR Premix Ex Taq (Takara, Dalian, China), 0.5 μl of DNA, 0.5 μl of each upstream and downstream primers, up to 10 μl with ddH_2_O (32). The data was analyzed by 2^−Δ*ΔCt*^ method (33).

### InDel genotyping of the *SNX29* gene

After testing the designed primers with DNA mixing pools, PCR amplification was performed in 13 μl of PCR reaction mixture, containing 6.5 μl of 2 × Taq Master mix (BioLinker, Shanghai, China), 0.5 μl of F and R primers, 0.5 μl of genomic DNA, and finally made up to 13 μl with ddH_2_O. The touch-down PCR procedure was utilized for PCR amplification (34, 35). Then PCR amplification products (P1 = 144 bp, P2 = 199 bp) were detected by 2% agarose gel electrophoresis.

### CNV genotyping of the *SNX29* gene

The primers were tested to ensure the that designed primers could amplify the target fragment (Supplementary Figure S1). Then, a total of 1,402 samples were used for testing. The total qPCR reaction system consisted of 10 μl comprised 5 μl of 2 × SYBR Premix Ex Taq (Takara, Dalian, China), 0.5 μl of DNA, 0.5 μl of each primer, and 3.5 μl of ddH_2_O. Previously explored experimental procedures were as follows, 95°C for 45 s, 40 cycles of 15 s at 95°C and 45 s at 60°C. Finally, the result was processed by method 2*2^−Δ*Ct*^·2*2^−Δ*Ct*^>2 was Gain, it < 2 was Loss, it = 2 was Median genotype (29).

### Statistical analyses

The genotype and allele frequencies, heterozygosity (He), homozygosity (Ho), polymorphism information content (PIC), Hardy-Weinberg equilibrium (HWE) and linkage disequilibrium (LD) were calculated using the SHEsis platform (http://analysis.bio-x.cn). Furthermore, analysis of variance (ANOVA) and χ^2^ test were used to explore the association between the variants and litter size in SPSS 26.0 (IBM, USA), when *P* < 0.05, all results were correlated with litter size, and all statistical tests were conducted by two-sided tests. The line model was reference previous study (36).

## Results

### The mRNA expression levels of the goat *SNX29* gene

**Figure 1:**
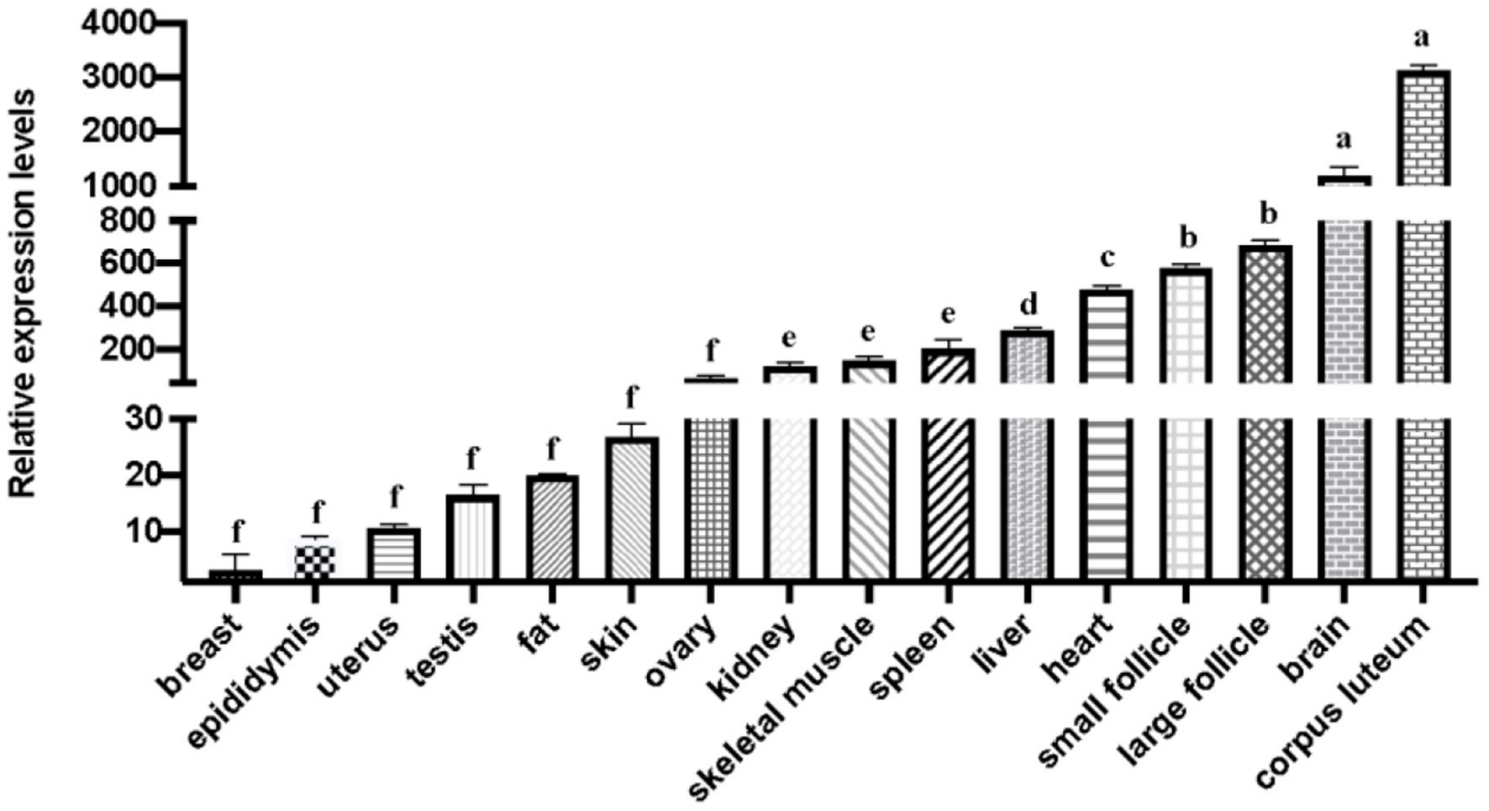
The mRNA expression of the goat *SNX29* gene. The values with different letters (a, b, d, e, f) indicates differ significantly at *P* < 0.05.

The results of the gene expression profile results indicated that the mRNA expression level of *SNX29* was higher in small follicles, large follicles and corpus luteum than in other tissues. Among 16 tissues, the relative expression level of the corpus luteum was the highest. In addition, more abundant expression was found in large and small follicles (Figure 1). Thus, this result suggests that the *SNX29* gene play an important role in goat follicle development.

### InDel detection: Genotype frequency, linkage disequilibrium and haplotype analyses of the goat *SNX29* gene

To further investigate the effect of *SNX29* on reproduction, we screened the genetic variations of *SNX29* and found that two InDel sites were polymorphic (P1-Del-17bp, P2-Del-20bp) among 11 pairs of primers. In the 17 bp deletion (*n* = 1,004), the frequency of ID genotype was much higher than that of II and DD. In the 20 bp deletion (*n* = 1,053), the number individuals with II genotype were much higher than the others. Besides, the P1-Del-17bp and P2-Del-20bp identified in the *SNX29* gene were not consistent the HWE (*P* < 0.05). According to PIC values, the detected *SNX29* P1-Del-17bp showed medium genetic diversity, while P2-Del-20bp exhibited low polymorphism in SBWC goats.

**Figure 2:**
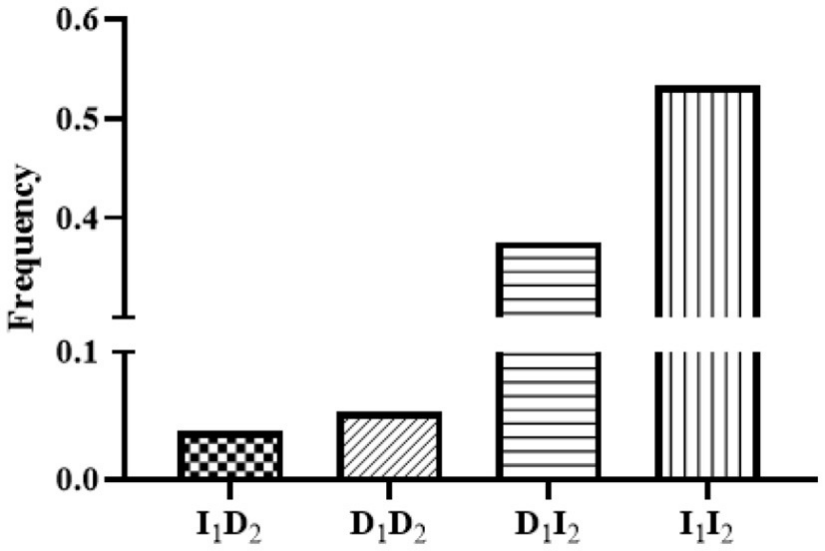
Haplotype frequencies of the two InDels within the goat *SNX29* gene. The 1 means P1-Del-17bp, 2 means P2-Del-20bp.

**Figure 3:**
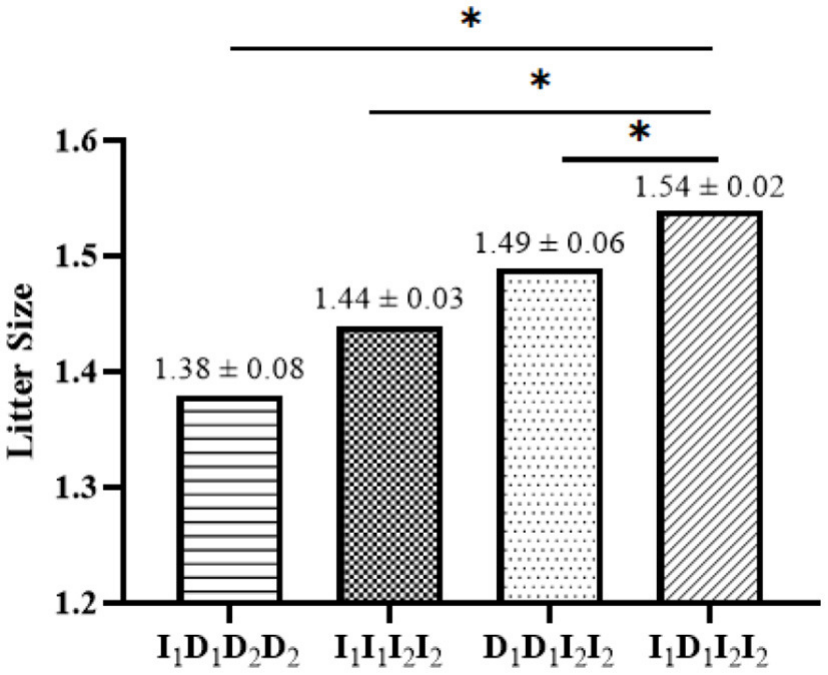
Least squares mean and standard error for litter size of different combination genotypes of the two InDels within the *SNX29* gene in SBWC goats. The 1 means P1-Del-17bp, 2 means P2-Del-20bp; The * means *P* < 0.05.

In the linkage disequilibrium (LD) analysis, the D' and *r*^2^ values for the linkage between P1-Del-17bp and P2-Del-20bp in the *SNX29* gene were 0.27 and 0.01, respectively, which indicated that these two polymorphisms were not strongly linked. The haplotype analysis results for the *SNX29* gene generated four haplotypes, and I_1_I_2_ had the highest frequency (Figure 2). Between the litter size and the two different combination genotypes of two InDels showed that they differed significantly (*P* < 0.05) in least-squares mean and standard error (Figure 3).

### CNV detection: Frequency of the goat *SNX29* gene genotypes

There were five CNV loci in the goat genome, and four of them (CNV1, CNV3, CNV4, CNV5) showed three variant types, while CNV2 had one variant type in SBWC goats. In four loci, the individual number of Gain genotype (CNV1 = 214, CNV3 = 239, CNV4 = 133, CNV5 = 197) was higher than Loss and Median, and the Gain frequencies in four sites (CNV1 = 0.834, CNV3 = 0.872, CNV4 = 0.516, CNV5 = 0.563) were significantly higher (Table 2).

**Table 2:** Typical frequencies of copy number variations within the *SNX29* gene in SBWC goats.

**CNV loci**	**Sizes**	**Typic frequencies**		
		**Loss**	**Median**	**Gain**
CNV1	*n* = 254	0.063 (*n* = 16)	0.094 (*n* = 24)	0.843 (*n* = 214)
CNV2	*n* = 266	0 (*n* = 0)	0.008 (*n* = 2)	0.992 (*n* = 264)
CNV3	*n* = 274	0.084 (*n* = 23)	0.044 (*n* = 12)	0.872 (*n* = 239)
CNV4	*n* = 258	0.186 (*n* = 48)	0.298 (*n* = 77)	0.516 (*n* = 133)
CNV5	*n* = 350	0.126 (*n* = 44)	0.311 (*n* = 109)	0.563 (*n* = 197)

### Association analyses of InDel and CNV of the goat *SNX29* gene

**Figure 4:**
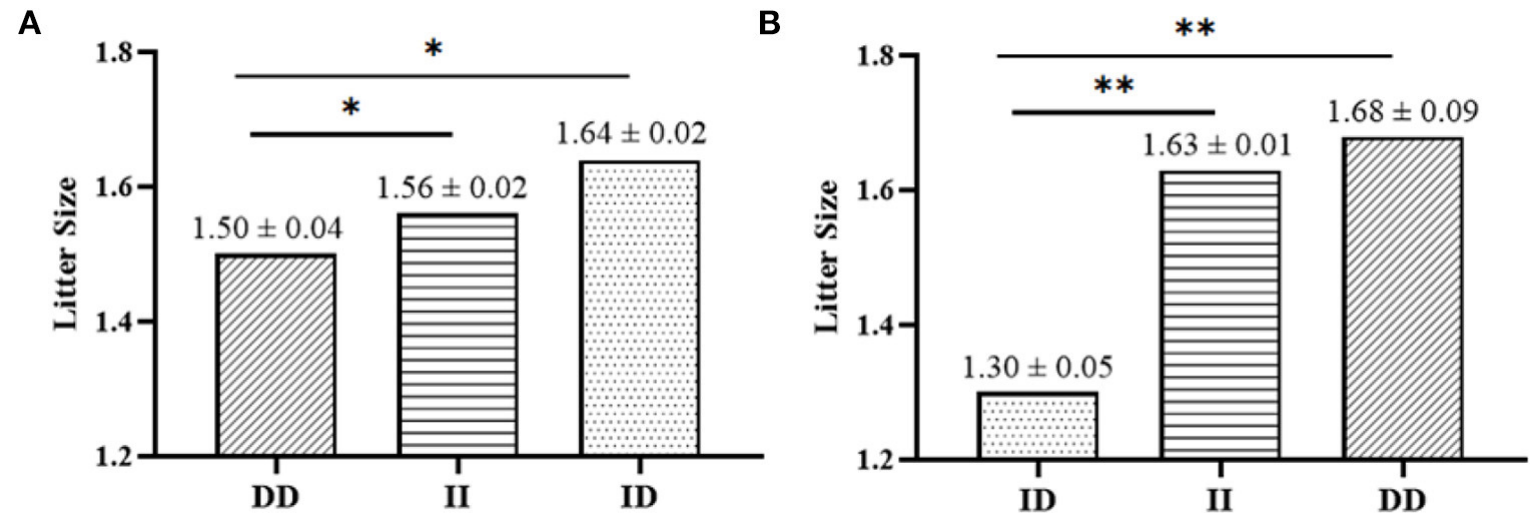
Association analyses between litter size and the two InDels in SBWC goats; **(A)** P1-Del-17bp, **(B)** P2-Del-20bp. * means *P* < 0.05; ** means *P* < 0.01.

**Figure 5:**
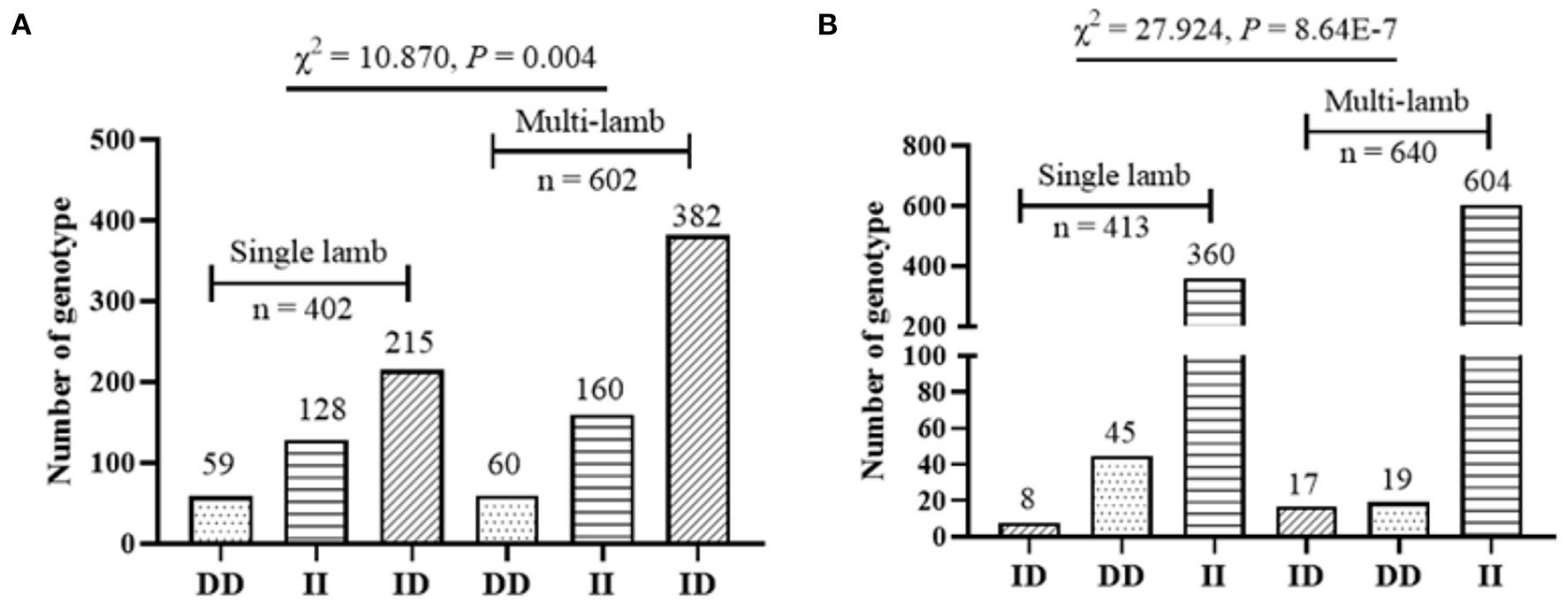
Genotype distribution between mothers of single-lamb and multi-lamb in SBWC goats; (**A)** P1-Del-17bp; **(B)** P2-Del-20bp.

It has been found that two InDel loci were related to litter size by association analyses. The 17 bp deletion (*P* = 0.022) and the 20 bp deletion (*P* = 1.000E-6) within the *SNX29* gene were discovered to be significantly associated with litter size, in which individuals the DD genotype of P2-Del-20bp (*P* < 0.01) and the ID genotype of P1-Del-17bp (*P* < 0.05) had larger litter size (Table 3; Figure 4). Moreover, the χ^2^ test showed that the genotype distribution of P1-Del-17bp (χ^2^ =10.870, *P* = 0.004) and P2-Del-20bp (χ^2^ = 27.924, *P* = 8.640E-7) were distinct between mothers of single-lamb and multi-lamb in 2,057 SBWC goats (Table 4; Figure 5).

**Table 3:** Association analyses between litter size and the two InDels in SBWC goats.

**InDel loci**	**Genotype**	**Typic frequencies (AVG ±SE)**	***P*-values**
P1-Del-17bp	II	1.56 ± 0.02^a^ (*n* = 288)	**0.022**
	ID	1.64 ± 0.02^a^ (*n* = 597)	
	DD	1.50 ± 0.04^b^ (*n* = 119)	
P2-Del-20bp	II	1.63 ± 0.01^A^ (*n* = 964)	**1.000E-6**
	ID	1.30 ± 0.05^B^ (*n* = 64)	
	DD	1.68 ± 0.09^A^ (*n* = 25)	

*Values with different letters (A, B/a, b) within the same row differ significantly at P < 0.01/P < 0.05. AVG means average, SE means standard error. The bold values indicates the value of P < 0.05*.

**Table 4:** Genotype distribution between mothers of single-lamb and multi-lamb in SBWC goats.

**InDel loci**	**Genotypes**	**Single-lamb**	**Multi-lamb**	**Total**	**Independent χ^2^ *P*-values**
P1-Del-17bp	II	128	160	288	χ^2^ =10.870 df = 2, *P* = 0.004
	ID	215	382	597	
	DD	59	60	119	
P2-Del-20bp	II	360	604	964	χ^2^ = 27.924 df = 2, *P* = 8.640E-7
	ID	8	17	25	
	DD	45	19	64	

**Figure 6:**
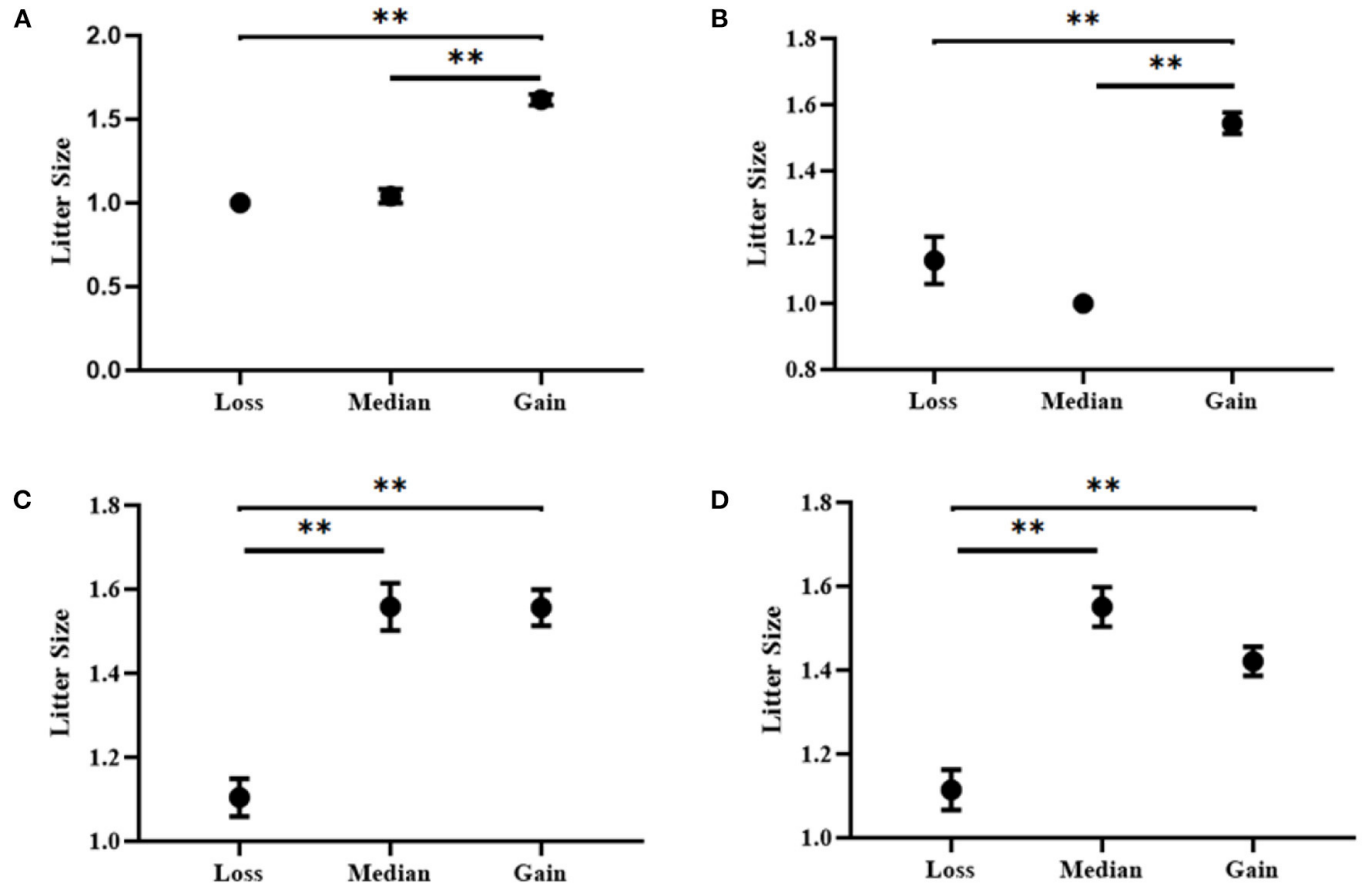
Association analyses between litter size and copy number variation types in SBWC goats. **(A)** CNV1; **(B)** CNV3; **(C)** CNV4; **(D)** CNV5. ** means *P* < 0.01.

Additionally, four CNV loci also had significant correlations with litter size (CNV1 = 3.997E-15, CNV3 = 2.900E-5, CNV4 = 7.387E-11, CNV 5 = 8.332E-9) in our detected population (*n* = 1,402). In CNV5 individuals with median genotype were superior to those with loss or Gain genotypes in term of litter size, and in the other three CNVs, the gain genotype performed better reproductive traits (Table 5; Figure 6). Besides, the χ^2^ analysis results demonstrated the two groups of goats with different litter types were significantly different (*P* < 0.01) (Table 6).

**Table 5:** Association analyses between litter size and copy number variation types in SBWC goats.

**CNV loci**	**Typic frequencies (AVG** ±**SE)**			***P*-values**
	**Loss**	**Median**	**Gain**	
CNV1	1.00 ± 0^B^ (*n* = 16)	1.04 ± 0.04^B^ (*n* = 24)	1.62 ± 0.03^A^ (*n* = 214)	**3.997E-15**
CNV3	1.13 ± 0.07^B^ (*n* = 23)	1.00 ± 0^B^ (*n* = 12)	1.54 ± 0.03^A^ (*n* = 239)	**2.900E-5**
CNV4	1.10 ± 0.04^B^ (*n* = 48)	1.56 ± 0.06^A^ (*n* = 77)	1.56 ± 0.04 ^A^ (*n* = 133)	**7.384E-11**
CNV5	1.11 ± 0.05^B^ (*n* = 44)	1.55 ± 0.05^A^ (*n* = 109)	1.42 ± 0.04^A^ (*n* = 197)	**8.332E-9**

*Values with different letters (A, B) within the same row differ significantly at P < 0.01. AVG means average, SE means standard error*.

**Table 6:** The distribution of different CNVs between mothers of single-lamb and multi-lamb population.

**CNV loci**	**Litter size**	**Loss**	**Median**	**Gain**	**Independent χ^2^, *P*-values**
CNV1	Single lamb	16	23	83	χ^2^ = 46.612, df = 2, *P* = 2.204E-13
	Multi-lamb	0	1	131	
CNV3	Single lamb	20	12	109	χ^2^ = 26.199, df = 2, *P* = 1.389E-7
	Multi-lamb	3	0	130	
CNV4	Single lamb	43	34	59	χ^2^ = 32.162, df = 2, *P* = 5.377E-8
	Multi-lamb	5	43	74	
CNV5	Single lamb	39	49	114	χ^2^ = 24.513, df = 2, *P* = 4.000E-6
	Multi-lamb	5	60	83	

## Discussion

To explore the function of the *SNX29* gene, the mRNA expression of this gene was first detected in different goat tissues, and we found that it was highly expressed in the corpus luteum and other reproductive organs. Previous studies have shown that *SNX24* regulates the level of estrogen (9). These results suggested that the *SNX29* gene may have a fertility-related function and plays a crucial role in reproductive performance.

As the main types of genetic variations, InDels and CNVs have contributed greatly to MAS. Although previous studies have reported that CNV27 in the *SNX29* gene significantly affects the growth traits in goats (37), and that the two InDels within this gene are significantly correlated with chest width, chest depth, chest circumference and hip width in goats (30), the relationship between InDel and CNV and litter size of goats has not been studied. Thus, in this study, 11 InDel loci and five CNV loci were retrieved from the database. The results showed that only two InDel loci and four CNV loci were polymorphic in SBWC goats, whereas other loci exhibited only a single mutation type. Despite the fact that all variants are located in intronic regions, the mutations are located at non-coding regions on chromosomes may affect the binding of transcription factors (38).

Furthermore, the result of the LD analysis showed no linkage between the two InDel loci, which indicates that the two mutation loci are physically far apart on the chromosomes (39). Haplotype frequencies and different genotype combinations of the two InDels were significant in goats. Besides, the PIC of P2-Del-20bp exhibited low polymorphism and the PIC of P1-Del-17bp showed medium polymorphism, indicating that their solid potential value for selection in goat breeds (40). Moreover, these variants did not conform to HWE. This could be due to the fact that artificial intervention results in individuals not being able to naturally mate with each other. We also found that the two InDel loci had multiple genotypes. Interestingly, the individual number of three genotypes was not equal. The individuals with ID genotype were more than those with II and DD genotype in P1-Del-17bp. The number of II genotypes was more than the ID and DD genotype in P2-Del-20bp. Many factors can change the frequency of the three genotypes, such as isolation, genetic drift and directional mating (41). These outcomes suggest that the ID genotype of 17 bp deletion and the II genotype of 20 bp InDel have a positive effect on litter size (42). Meanwhile, in CNV5, individuals with median genotype were superior than those with loss or gain genotypes in term of litter size, and in other three CNVs, the gain genotype showed better reproductive traits, which could be due to genetic drift migration, mutation, gene recombination, and selection (43). Thus, we concluded that the individuals with the Gain genotype perform better in reproductive performance, and more individuals with Gain genotype are preserved in breeding. The InDel loci and CNV loci may also have a remarkable influence on the *SNX29* mRNA expression in the corpus luteum, small follicles and large follicles. Therefore, further researches should be conducted to explore the relationship between variants and reproduction.

The association between the *SNX29* gene variants and litter size was analyzed. Notably, association analysis showed that two InDel loci and four CNV loci were observably associated with the litter size (*P* < 0.01; *P* < 0.05), which supports our conjecture, while no previous studies have demonstrated that the *SNX29* gene is involved in goat reproduction. Referring to the relevant literature, some genes have affected both neural and reproductive development in animals. For instance, it has been reported that *DSCAML1* affects both neural development and reproductive traits in animals (44, 45). At the same time, growth traits and reproductive traits are inextricably linked. Some genes play a role in both reproductive traits and growth traits in livestock. For instance, the mutation of the *PLAG1* gene has affected growth traits and reproductive traits in animals (21–23). Although the mechanisms of these genes that simultaneously affect growth, nerves, reproduction are not well understood, we speculated that the *SNX29* gene also has the same function as the aforementioned-genes, which are involved in the development of animal growth, nerve, reproduction and other traits.

Collectively, goat *SNX29* mRNA expression in the corpus luteum, small follicles and large follicles significantly differed from other tissues. Two InDels (P1-Del-17bp and P2-Del-20bp) and four CNVs (CNV1, CNV3, CNV4 and CNV5) were significantly association with litter size. This finding reveals the reproductive function of the *SNX29* gene in livestock, and the InDel and CNV variations of the *SNX29* gene in this study will make the use of MAS more widespread in goat breeding, providing a theoretical guidance for goat breed selection.

## Conclusion

The *SNX29* gene was highly expressed in the corpus luteum and other reproductive tissues, and the *SNX29* gene loci (InDels and CNVs) were associated with litter size in SBWC goats. Thus, this gene may be an essential functional candidate gene for reproductive traits.

## Data availability statement

The original contributions presented in the study are included in the article/Supplementary material, further inquiries can be directed to the corresponding authors.

## Ethics statement

The animal study was reviewed and approved by Northwest A&F University.

## Author contributions

Sample collection: QW, YB, ZW, and HZ. Experimental operation: QW, YB, and ZW. Data collation and analysis: QW, YB, HZ, and XW. Article writing: QW. Paper revision and editing: QW, YB, CP, and XW. Project management: CP, XW, and ML. All authors contributed to the article and approved the submitted version.

## Funding

This work was supported by the National Natural Science Foundation of China [No. 32002166].

## Conflict of interest

The authors declare that the research was conducted in the absence of any commercial or financial relationships that could be construed as a potential conflict of interest.

## Publisher's note

All claims expressed in this article are solely those of the authors and do not necessarily represent those of their affiliated organizations, or those of the publisher, the editors and the reviewers. Any product that may be evaluated in this article, or claim that may be made by its manufacturer, is not guaranteed or endorsed by the publisher.
