# Supplementary figures and images for: Goat SNX29: mRNA expression, InDel and CNV detection, and their associations with litter size
Source: Front Vet Sci. 2022 Aug 10;9:981315. doi: 10.3389/fvets.2022.981315 (PMC9399746; doi:10.3389/fvets.2022.981315)

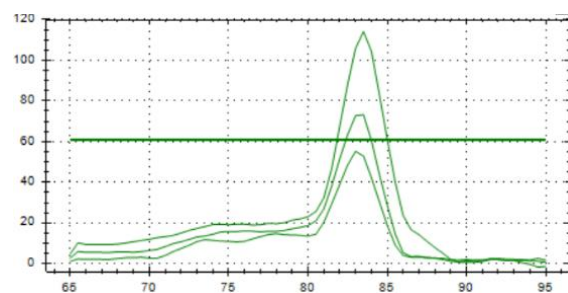

**Figure S1.** The melting curve of CNV1.

Supplement: Supplementary file 1 [file Image_1.pdf]

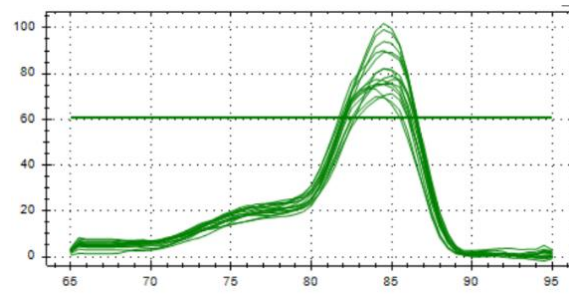

**Figure S2.** The melting curve of CNV2.

Supplement: Supplementary file 2 [file Image_2.pdf]

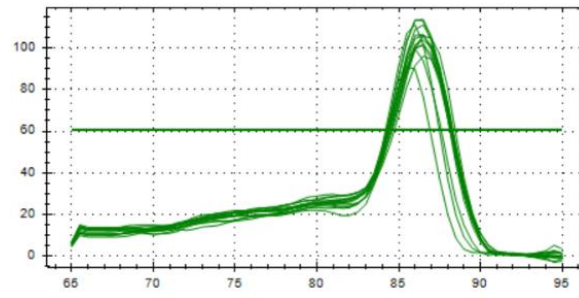

**Figure S3.** The melting curve of CNV3.

Supplement: Supplementary file 3 [file Image_3.pdf]

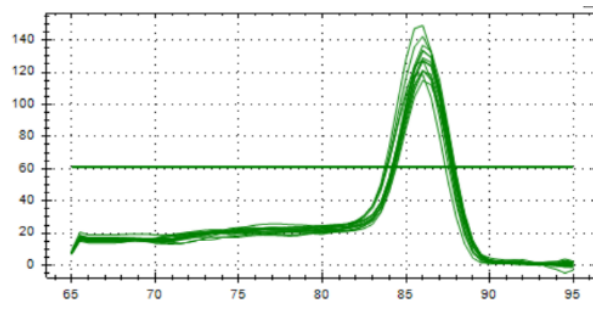

**Figure S4.** The melting curve of CNV4.

Supplement: Supplementary file 4 [file Image_4.pdf]

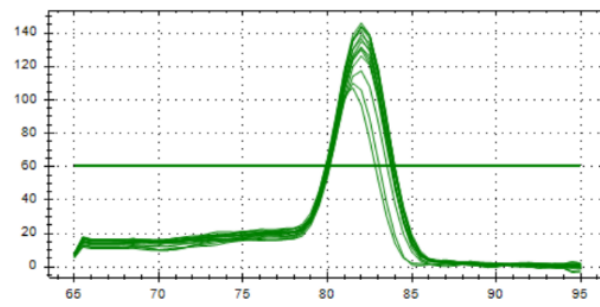

**Figure S5.** The melting curve of CNV5.

Supplement: Supplementary file 5 [file Image_5.pdf]
