# Supplementary material for: Goat SNX29: mRNA expression, InDel and CNV detection, and their associations with litter size
Source: Front Vet Sci. 2022 Aug 10;9:981315. doi: 10.3389/fvets.2022.981315 (PMC9399746; doi:10.3389/fvets.2022.981315)
Supplement: Supplementary file 6 [file Table_1.docx]

**Table S1.** The primer information of InDels.

| Variant loci | Variant number | Variant sequence | Variant length | Chromosomal location | Variant type | Variant position |
| --- | --- | --- | --- | --- | --- | --- |
| P1-Del-17bp | Rs659002477 | TAAAGGAAAGCAATGTA/- | 17 bp | 25: 10559298-10559314 | Deletion | Upstream |
| P2-Del-20bp | Rs654310334 | AGCTTCCGGTGAGCCTGTCG/- | 20 bp | 25: 10918982-10919002 | Deletion | Intron |
| P3-Del-20bp | Rs666003090 | CCCCTGCCTGTTGCCTCCAG/- | 20 bp | 25: 10750295-10750314 | Deletion | Intron |
| P4-Del-18bp | Rs646325249 | TCAGTCTTTCACCACTGA/- | 18 bp | 25: 10775771-10775788 | Deletion | Intron |
| P5-Del-15bp | Rs651194239 | GGCTCCTAGTCACCC/- | 15 bp | 25: 10885504-10885518 | Deletion | Intron |
| P6-Del-20bp | Rs646849071 | TGGGTTGCTTTAGGATGCTT/- | 20 bp | 25: 11025845-11025864 | Deletion | Intron |
| P7-Del-18bp | Rs636385443 | AATGTTTACCAAAAAAGG/- | 18 bp | 25: 11031301-11031318 | Deletion | Intron |
| P8-In-16bp | Rs667270910 | -/GTTGCATTAGTCATGG | 16 bp | 25: 11080502 & 11080503 | Insertion | Intron |
| P9-Del-15bp | Rs656101837 | TCACCTGGAATGTGA/- | 15 bp | 25: 11087210-11087224 | Deletion | Intron |
| P10-Del-18bp | Rs668089309 | TTTTATTTTTTTATTTTC/- | 18 bp | 25: 11149709-11149726 | Deletion | Intron |
| P11-In-8bp | Rs652704732 | -/GGCAGGAT | 8 bp | 25: 11163269 & 11163270 | Insertion | Downstream |

**Note:** Variant number were from Ensembl.
