# Supplementary material for: Goat SNX29: mRNA expression, InDel and CNV detection, and their associations with litter size
Source: Front Vet Sci. 2022 Aug 10;9:981315. doi: 10.3389/fvets.2022.981315 (PMC9399746; doi:10.3389/fvets.2022.981315)
Supplement: Supplementary file 7 [file Table_2.docx]

**Table S2.** The primer information of CNVs.

| Variant loci | Chromosomal location | Variant length | Variant position |
| --- | --- | --- | --- |
| CNV1 | 25: 10565201-10566800 | 1600 | Intron |
| CNV2 | 25: 10606001-10608000 | 2000 | Intron |
| CNV3 | 25: 10674401-10676000 | 1600 | Intron |
| CNV4 | 25: 10808001-10810000 | 2000 | Intron |
| CNV5 | 25: 10832401-10834800 | 2400 | Intron |
